# The Role of Transcranial Direct Current Stimulation in Chronic Shoulder Pain: A Scoping Review

**DOI:** 10.3390/brainsci15060584

**Published:** 2025-05-28

**Authors:** Roberto Tedeschi, Federica Giorgi, Danilo Donati

**Affiliations:** 1 Independent Researcher, 40100 Bologna, Italy; 2 Pediatric Physical Medicine and Rehabilitation Unit, IRCCS Institute of Neurological Sciences, 40139 Bologna, Italy; 3 Physical Therapy and Rehabilitation Unit, Policlinico di Modena, 41125 Modena, Italy; 4 Clinical and Experimental Medicine PhD Program, University of Modena and Reggio Emilia, 41121 Modena, Italy

**Keywords:** shoulder pain, transcranial direct current stimulation, chronic pain, musculoskeletal disorders, central sensitization

## Abstract

Background: Chronic shoulder pain is a prevalent musculoskeletal disorder often associated with central sensitisation, which limits the effectiveness of conventional therapies. Transcranial direct current stimulation (tDCS) has emerged as a non-invasive neuromodulatory intervention to modulate cortical excitability and potentially improve pain and functional outcomes. Methods: This scoping review followed the Joanna Briggs Institute (JBI) framework and PRISMA-ScR guidelines. A systematic search was conducted across MEDLINE, CENTRAL, Scopus, PEDro, and Web of Science to identify studies evaluating the effects of tDCS on pain and function in adults with rotator cuff disorders, myofascial pain syndrome (MPS), or subacromial pain syndrome (SAPS). Data were extracted and synthesised qualitatively. Results: Four studies met the inclusion criteria. tDCS demonstrated variable efficacy: some trials reported no additional benefit when used alongside corticosteroid injections or sensorimotor training (e.g., SAPS and rotator cuff tendinopathy), while others showed enhanced pain reduction and functional gains, particularly in MPS. Targeting the dorsolateral prefrontal cortex (DLPFC) appeared more effective than stimulating the primary motor cortex (M1) in modulating pain. Functional improvements were generally observed, though not consistently superior to sham interventions. Conclusions: Preliminary evidence suggests that tDCS may represent a promising adjunctive treatment for chronic shoulder pain, particularly in MPS. However, due to the limited number of studies and heterogeneity in methods, conclusions should be interpreted with caution. However, heterogeneity in study protocols, stimulation targets, and patient populations limits conclusive recommendations. Standardised protocols and larger trials are needed to determine the optimal application of tDCS in clinical shoulder pain management.

## 1. Introduction

Shoulder pain is a common musculoskeletal complaint affecting a significant proportion of the adult population, with rotator cuff disorders, myofascial pain syndrome (MPS), and subacromial pain syndrome (SAPS) among the leading causes [1,2]. Orthopaedic shoulder pain often arises from degenerative, inflammatory, or traumatic aetiologies that impair the functionality of the shoulder joint, leading to disability and a reduced quality of life [3]. Historically, tDCS has been investigated in various musculoskeletal and neurological pain conditions, including knee osteoarthritis, low-back pain, and complex regional pain syndrome. Its use has also been explored in reducing secondary motor impairment due to cortical inhibition, with the aim of restoring postural control and improving functional capacity. Moreover, cathodal stimulation of the contralateral cortex has been proposed to enhance inter-hemispheric balance, although its application in shoulder pain is still limited. Despite advances in conservative and surgical management, a substantial number of patients experience persistent pain and functional limitations post-treatment [3,4,5,6]. Conventional treatments for orthopaedic shoulder pain include physical therapy [7,8,9,10], pharmacological interventions [11], and corticosteroid injections (CSIs). While these approaches provide symptomatic relief, they may not directly address central sensitisation mechanisms that can persist in some patients with prolonged nociceptive input. However, it is acknowledged that central sensitisation may gradually subside if peripheral drivers are removed or significantly reduced. In addition, chronic shoulder pain is often accompanied by secondary changes in soft tissues and joint mechanics due to long-term disuse or movement avoidance [7,11,12,13]. Central sensitization is a pathological condition where the central nervous system (CNS) exhibits an exaggerated response to stimuli due to maladaptive plasticity changes [14,15,16]. Emerging evidence suggests that non-invasive brain stimulation techniques, such as transcranial direct current stimulation (tDCS), may play a pivotal role in modulating central sensitization and improving pain outcomes in musculoskeletal disorders [17,18,19]. It is important to distinguish nociceptive from nociplastic pain pathways when considering neuromodulatory interventions. While conditions like rotator cuff tendinopathy and SAPS are primarily nociceptive, MPS is increasingly recognised as a nociplastic pain condition, where central sensitisation and altered cortical processing play a dominant role. This difference may underlie the variable response to tDCS observed across shoulder pain subtypes. Transcranial direct current stimulation is a neuromodulatory technique that involves the application of a low-intensity electrical current to specific cortical areas, aiming to modulate cortical excitability. In this context, “neuromodulation” refers broadly to the use of non-invasive electrical stimulation to alter central nervous system activity. “Cortical modulation” denotes the immediate influence on neuronal excitability, while “neuroplasticity” describes longer-term changes in synaptic strength or brain structure induced by repeated stimulation. These concepts are interrelated and collectively underpin the theoretical rationale for tDCS in chronic pain management. The motor cortex, particularly the primary motor cortex (M1), has been identified as a target for tDCS in pain management due to its role in descending pain modulation pathways [20,21,22,23]. In contrast, stimulation of the dorsolateral prefrontal cortex (DLPFC) has shown promise in modulating pain through top–down cognitive and emotional pathways, especially in patients with nociplastic pain profiles. The DLPFC is also implicated in attentional and affective components of pain, making it a relevant target in disorders such as MPS. Recent clinical studies have demonstrated that anodal tDCS over M1 can reduce pain intensity and improve functional outcomes in patients with chronic pain conditions, including low-back pain, fibromyalgia, and myofascial pain [8,9,24,25,26]. However, there is limited research exploring the efficacy of tDCS in the management of orthopaedic shoulder pain. The current literature on tDCS and shoulder pain is sparse but promising. Larrivée et al. (2021) [27] conducted a randomized controlled pilot study investigating the effects of anodal tDCS following subacromial corticosteroid injections in patients with SAPS. The study found that while all groups showed significant improvement in pain and function, a single session of tDCS did not result in additional benefits compared to sham tDCS or no intervention. Similarly, Belley et al. (2018) [28] explored the use of tDCS combined with sensorimotor training in individuals with rotator cuff tendinopathy, concluding that while both groups improved, the addition of tDCS did not enhance treatment outcomes [28]. Conversely, studies in myofascial pain syndrome, such as those by Choi et al. (2014) [29] and Sakrajai et al. (2014) [30], have shown that tDCS can augment traditional treatments like trigger point injections and physical therapy, resulting in more substantial pain reduction [29,30]. Given the potential role of tDCS in modulating central pain mechanisms and the mixed findings from current studies, there is a pressing need to systematically explore its application in orthopaedic shoulder pain management. This scoping review aims to address this gap by synthesizing existing evidence on the use of tDCS for orthopaedic shoulder pain, with a focus on rotator cuff disorders, MPS, and SAPS. The growing burden of chronic orthopaedic shoulder pain necessitates innovative approaches to pain management. While traditional interventions target peripheral pain mechanisms, tDCS offers a novel avenue for addressing central pain pathways. This review will provide a critical analysis of the current evidence and propose future directions for integrating tDCS into clinical practice for the management of orthopaedic shoulder pain.

## 2. Methods

This scoping review was conducted in accordance with the methodological approach proposed by the Joanna Briggs Institute (JBI) for scoping reviews [31]. To enhance the clarity and completeness of the reporting, the review adhered to the PRISMA-ScR (Preferred Reporting Items for Systematic Reviews and Meta-Analyses extension for Scoping Reviews) checklist [32]. This review did not have a registered protocol, as registration is not mandatory for scoping reviews according to JBI and PRISMA-ScR guidelines.

### 2.1. Review Question

We formulated the following research question: “What is the impact of transcranial direct current stimulation (tDCS) on pain reduction and functional recovery in orthopedic shoulder conditions?”

### 2.2. Eligibility Criteria

Studies were considered for inclusion based on the Population, Concept, and Context (PCC) framework criteria.

#### 2.2.1. Population

Studies included adults aged 18 years and older experiencing orthopaedic shoulder pain. This population includes individuals diagnosed with the folowing:

- Rotator cuff disorders, such as tendinopathy or tears.
- Myofascial pain syndrome (MPS) in the shoulder region, characterized by trigger points and referred pain.
- Subacromial pain syndrome (SAPS), including subacromial bursitis and impingement syndromes.

Exclusion criteria included individuals with neurological disorders, systemic inflammatory conditions (e.g., rheumatoid arthritis), or prior surgical interventions on the affected shoulder within six months.

#### 2.2.2. Concept

The primary concept explored in this review is the use of transcranial direct current stimulation (tDCS) as a therapeutic intervention for pain management. Studies were included for the following reasons:

- They investigated the efficacy of tDCS in reducing pain intensity.
- They assessed improvements in functional recovery, such as range of motion, strength, and daily activity levels.
- They focused on tDCS applied over motor cortex regions relevant to shoulder pain (e.g., primary motor cortex M1).

Secondary outcomes, such as quality of life improvements, patient-reported satisfaction, and neurophysiological changes (e.g., cortical excitability), were also considered.

#### 2.2.3. Context

This review focused on clinical settings where tDCS was used as part of rehabilitation or pain management protocols. These settings include the following:

- Physical therapy clinics.
- Pain management centres.
- Research laboratories conducting experimental neuromodulation studies.

### 2.3. Exclusion Criteria

Studies falling outside the predefined criteria of Population, Concept, and Context (PCC) were excluded from this review.

### 2.4. Search Strategy

An initial targeted search was carried out in MEDLINE (via PubMed) to identify relevant studies and refine the search terms. These terms were then used to build a complete strategy, which was adapted for CENTRAL, Scopus, PEDro, and Web of Science. The final search was completed on 23 December 2024, with no date restrictions. Full search strings for each database are provided in Supplementary File S1.

### 2.5. Data Extraction and Data Synthesis

Data extraction was carried out systematically using a standardised form to collect key information, including study design, population, interventions, outcomes, and main findings. The results were organised by outcome domains to enable comparison. A qualitative synthesis highlighted common themes, inconsistencies, and gaps, while quantitative data were summarised where relevant to identify trends. Data extraction was independently performed by two reviewers. Disagreements were resolved through discussion or consultation with a third reviewer. In accordance with the JBI methodology for scoping reviews, a formal risk-of-bias assessment was not conducted, as the primary aim was to map existing evidence rather than appraise the quality of individual studies. While risk-of-bias assessment is not mandatory in scoping reviews, we acknowledge its increasing value in strengthening the interpretation of results. Therefore, we qualitatively discussed the methodological strengths and limitations of included studies in the Section 4 and Section 5 to offer better insight into the evidence base.

## 3. Results

**Figure 1:**
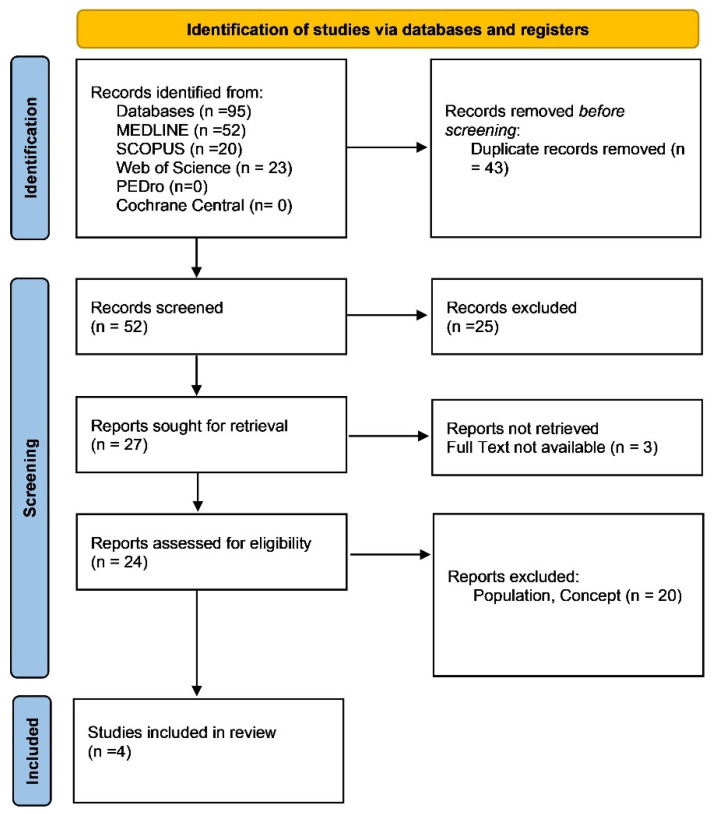
Preferred reporting items for systematic reviews and meta-analyses 2020 (PRISMA) flow diagram.

As presented in the PRISMA 2020 [32] flow diagram (Figure 1), from 95 records identified by the initial literature searches, 91 were excluded and 4 articles were included (Table 1). A detailed overview of the stimulation parameters, session characteristics, and clinical outcomes is reported in Table 2.

Table 2 also allows for comparative analysis of key stimulation variables—such as intensity, duration, and cortical target—offering insights into which configurations may yield more promising results.

### 3.1. Pain Reduction

Pain reduction was a significant outcome across all studies:

- Larrivée et al. (2021) [27]: Patients in both the real and sham tDCS groups experienced substantial pain relief, with a mean reduction of 3.5 points on the VAS in the real tDCS group and 3.3 points in the sham group. These reductions indicate that pain relief was primarily due to the corticosteroid injections rather than the tDCS intervention.
- Choi et al. (2014) [29]: In this study, pain intensity significantly decreased in the DLPFC group by an average of 2.5 points on the VAS. This reduction was observed after the second stimulation session and was maintained throughout the treatment period. The pain reduction in the M1 and sham groups was less pronounced, highlighting the potential role of targeting the DLPFC for chronic pain management.
- Belley et al. (2018) [28]: Pain-related disability, measured using the DASH score, improved from a baseline of 45.6 to 29.3 in both the real and sham tDCS groups after 12 weeks. The WORC index showed similar improvements, with both groups reporting significant reductions in shoulder-related disability. However, the study did not find any additional benefits of real tDCS over sham stimulation.
- Sakrajai et al. (2014) [30]: Patients in the active tDCS group reported a mean pain reduction of 4 points on the NRS, compared to a 2-point reduction in the sham group. Pain relief in the active tDCS group was more sustained, with improvements maintained at the 1-week and 4-week follow-ups.

Baseline and post-treatment pain scores (VAS) are summarised in Table 3 and Table 4 to illustrate absolute pain reductions across intervention and control groups.

### 3.2. Functional Recovery

Functional recovery was assessed using various tools:

- Larrivée et al. (2021) [27]: The SANE scores improved significantly, from an average of 65% to 85% post-treatment. Both the tDCS and sham groups showed similar levels of improvement, suggesting that the corticosteroid injection was the primary driver of functional recovery.
- Choi et al. (2014) [29]: Patients in the DLPFC group demonstrated better improvements in range of motion and pain thresholds compared to the M1 and sham groups. The study reported a 15% increase in functional recovery scores in the DLPFC group, highlighting the potential of tDCS to enhance physical performance.
- Belley et al. (2018) [28]: Functional outcomes were measured using the DASH and WORC indices. Both groups showed improvements from baseline to week 12, with the DASH score decreasing by approximately 16 points and the WORC score increasing by 25 points. However, no additional benefits were observed in the real tDCS group.
- Sakrajai et al. (2014) [30]: Shoulder adduction PROM improved by an average of 15 degrees in the active tDCS group, compared to a 7-degree improvement in the sham group. The active tDCS group also reported faster recovery times, with improvements seen as early as the second treatment session.

### 3.3. Cortical Modulation and Neuroplasticity

Two studies explored the impact of tDCS on cortical modulation:

While the reviewed studies [29,30] reported clinical improvements, they did not directly assess cortical excitability through objective neurophysiological or neuroimaging methods. However, Jog et al. (2023) [33] demonstrated that tDCS can induce structural plasticity, supporting the theoretical framework of cortical modulation in chronic pain rehabilitation. Recent neuroimaging research has also provided insight into the structural plasticity induced by tDCS. Jog et al. (2023) [33] demonstrated that tDCS can lead to measurable increases in grey matter volume in targeted cortical regions, using structural MRI. Furthermore, the use of high-definition tDCS (HD-tDCS) was associated with more focal stimulation and potentially greater neuromodulatory effects compared to conventional tDCS. These findings support the hypothesis that tDCS not only modulates cortical excitability but may also induce long-term structural changes in the brain, which could be beneficial in the context of chronic pain rehabilitation (Jog et al., 2023) [33].

### 3.4. Quality of Life and Patient Satisfaction

Although not a primary focus in most studies, improvements in quality of life and patient satisfaction were noted:

- Belley et al. (2018) [28]: The study reported an 85% patient satisfaction rate at the end of the 12-week intervention. Participants in both the real and sham tDCS groups reported positive outcomes, including reduced pain and improved shoulder function.
- Sakrajai et al. (2014) [30]: The study highlighted patient-reported outcomes such as decreased reliance on pain medications and increased satisfaction with the treatment. These findings suggest that tDCS may enhance patients’ overall treatment experience and quality of life.

A summary of outcomes stratified by clinical diagnosis and cortical target is presented in Table 3, highlighting differences in pain and function responses.

## 4. Discussion

The findings from this review highlight both the potential and limitations of transcranial direct current stimulation (tDCS) in managing orthopaedic shoulder pain. Across the included studies, tDCS demonstrated varying degrees of effectiveness in reducing pain intensity, enhancing functional recovery, and modulating cortical activity. However, the results were not consistently significant across different study designs and patient populations, which raises important questions about the optimal application of this intervention in clinical practice. One of the key insights from this review is the potential role of targeting different cortical areas to achieve specific outcomes. For instance, Choi et al. (2014) [29] demonstrated that stimulating the dorsolateral prefrontal cortex (DLPFC) resulted in more pronounced pain relief compared to stimulation of the primary motor cortex (M1). The DLPFC is involved in top–down modulation of pain and integrates cognitive–emotional processing, including attention, expectation, and affective components. These features might explain its potential superiority in conditions like MPS, which are closely associated with emotional distress and central sensitisation. This finding suggests that the choice of cortical target may be critical in optimizing the effects of tDCS for chronic pain conditions. The DLPFC is known to be involved in pain modulation through its connections with both the emotional and cognitive aspects of pain processing, which might explain the superior results observed in this study. In contrast, studies focusing on musculoskeletal conditions such as rotator cuff tendinopathy and subacromial pain syndrome (SAPS) reported mixed results regarding the added benefits of tDCS. Belley et al. (2018) [28] and Larrivée et al. (2021) [27] both observed significant improvements in pain and function across all study groups, but tDCS did not provide additional benefits over sham stimulation. These findings suggest that while tDCS may be beneficial in certain chronic pain conditions, its efficacy may be limited when used as an adjunct to conventional treatments like corticosteroid injections and physical therapy. Interestingly, the study by Sakrajai et al. (2014) [30] highlighted the potential for tDCS to enhance functional recovery, particularly in patients with myofascial pain syndrome (MPS). The active tDCS group showed greater improvements in shoulder range of motion (PROM) and sustained pain relief compared to the sham group. This finding aligns with the hypothesis that tDCS can induce neuroplastic changes that improve motor function and reduce pain perception. However, the differences in outcomes between musculoskeletal and myofascial pain syndromes underscore the need for a more nuanced approach to tDCS application, taking into account the underlying pathophysiology of the condition being treated. Overall, the heterogeneity of the results points to several areas that require further investigation. First, there is a need to standardise the stimulation protocols, including the choice of cortical target, stimulation intensity, and duration. Second, future research should explore the mechanisms underlying the differential effects of tDCS across various types of shoulder pain. Third, the role of patient-specific factors, such as baseline pain severity, psychological state, and responsiveness to neurostimulation, should be considered in designing personalized treatment protocols. Future studies should aim to harmonise tDCS parameters, including current intensity (e.g., 1–2 mA), session duration (e.g., 20 min), number of sessions (e.g., >5), and electrode montage (e.g., DLPFC vs. M1), to enhance comparability across studies. Beyond shoulder pain, tDCS has been applied to other orthopaedic pain syndromes such as chronic knee osteoarthritis, lateral epicondylalgia, and postoperative joint pain. In these contexts, tDCS has shown modest improvements in pain and function, particularly when combined with rehabilitation programs. These findings reinforce the notion that neuromodulation can be a useful adjunct in complex, multifactorial pain conditions when applied using well-defined parameters and patient selection criteria.

## 5. Limitations

Several limitations of the current evidence base must be acknowledged. The included studies varied significantly in their methodologies, patient populations, and outcome measures, making direct comparisons challenging. Additionally, the sample sizes in most studies were relatively small, which may limit the generalizability of the findings. Another important limitation is the lack of long-term follow-up data in many studies. While short-term benefits of tDCS have been demonstrated, the sustainability of these effects over time remains unclear. The variability in stimulation protocols, particularly the choice of cortical target and the parameters of tDCS application, further complicates the interpretation of the results. For instance, some studies targeted the primary motor cortex (M1), while others focused on the dorsolateral prefrontal cortex (DLPFC), which may account for some of the differences in outcomes. This lack of consistency highlights the need for more standardised protocols in future research. Finally, many of the studies relied on subjective measures of pain and function, which are prone to bias. Objective measures, such as neurophysiological assessments and imaging studies, could provide more robust evidence of the effects of tDCS on cortical modulation and pain pathways.

## 6. Clinical Practice Implications

Although the available evidence is preliminary and based on a small number of heterogeneous studies, some clinical implications can cautiously be drawn. tDCS may represent a promising adjunct in the multidisciplinary management of chronic shoulder pain, particularly for patients with myofascial pain syndrome (MPS), where central sensitisation and maladaptive neuroplasticity are likely contributors.

The studies reviewed suggest that tDCS, especially when targeting the dorsolateral prefrontal cortex (DLPFC), may support pain modulation through top–down emotional and cognitive pathways. However, these findings are exploratory and should not be interpreted as conclusive. Importantly, in musculoskeletal conditions such as rotator cuff tendinopathy and SAPS, the results were inconsistent, and no additional clinical benefit of tDCS over conventional care (e.g., corticosteroid injection or physical therapy) was demonstrated.

Therefore, tDCS should not be considered a standalone treatment for shoulder pain. Instead, it may be incorporated as a complementary tool within a multimodal strategy that includes exercise therapy, pharmacological support, and education. Clinicians should tailor the application of tDCS based on the individual’s pain mechanism and psychosocial profile.

Further research, including larger clinical trials and mechanistic studies, is needed before tDCS can be routinely recommended in clinical practice. Future work should also explore optimal stimulation parameters and patient selection criteria to better define tDCS’s role in orthopaedic pain rehabilitation [17,19,21].

## 7. Conclusions

This scoping review highlights the exploratory role of tDCS in the management of chronic orthopaedic shoulder pain. While some studies reported positive outcomes in myofascial pain syndrome, the results were inconsistent in other conditions such as rotator cuff tendinopathy and subacromial pain syndrome. The available evidence is based on small-sample trials with variable stimulation parameters and lacks robust clinical standardisation. This review suggests that future research should prioritise larger, methodologically rigorous trials, including detailed patient stratification and standardised tDCS protocols. Until then, tDCS may be considered an experimental, adjunctive option in selected patients with persistent shoulder pain not responsive to conventional therapies.

## Figures and Tables

**Table 1:** Summary of key studies on tDCS and shoulder pain.

Author, Year, and Study Type	Methods	Results	Outcomes Achieved
Larrivée et al., 2021 (Randomized Controlled Pilot Study) [27]	A total of 38 patients with SAPS received corticosteroid injections followed by either real, sham, or no tDCS. Pain and function were measured using the VAS and SANE.	All groups showed significant improvement in pain and function. No additional benefits of tDCS were observed compared to sham or no intervention.	Improvement in pain and upper limb function was observed; however, tDCS did not provide additional benefits.
Choi et al., 2014 (Randomized, Single-Blind Trial) [29]	A total of 21 patients with MPS were randomized into three groups to receive TPI and tDCS over different brain areas (M1, DLPFC). Pain was assessed using the VAS and the McGill Pain Questionnaire.	Pain reduction was observed in all groups, with significant changes in the DLPFC group starting after the second session.	Significant pain reduction in the DLPFC group was observed; tDCS showed promise as an adjunct to TPI.
Belley et al., 2018 (Triple-Blind Randomized Controlled Trial) [28]	A total of 40 patients with rotator cuff tendinopathy were divided into two groups receiving sensorimotor training with either real or sham tDCS. Outcomes were measured using DASH and WORC.	Both groups showed improvements in DASH and WORC scores over 12 weeks. No added effects of tDCS were found.	Functional improvements in both groups were found; no additional benefits of tDCS were noted.
Sakrajai et al., 2014 (Randomized Controlled Study) [30]	A total of 31 patients with chronic myofascial pain were randomized to receive either active or sham tDCS over M1 combined with standard care. Pain intensity and PROM were measured.	The active tDCS group reported greater reductions in pain intensity and improvement in shoulder adduction PROM compared to the sham group.	Pain intensity reduction and improvements in PROM were achieved faster in the active tDCS group.

Legend: DASH: Disabilities of the Arm, Shoulder, and Hand questionnaire; DLPFC: dorsolateral prefrontal cortex; M1: primary motor cortex; MPS: myofascial pain syndrome; PROM: passive range of motion; SANE: Single Assessment Numeric Evaluation; SAPS: subacromial pain syndrome; tDCS: transcranial direct current stimulation; TPI: trigger point injection; VAS: Visual Analog Scale; WORC: Western Ontario Rotator Cuff index.

**Table 2:** Stimulation parameters and clinical outcomes of included tDCS studies for shoulder pain.

Author, Year	Diagnosis	Sample Size	Anode/Cathode Montage	Current Intensity (mA)	Session Duration (min)	No. of Sessions	Follow-Up Period	Main Findings
Larrivée et al., 2021 [27]	SAPS	38	M1/Contralateral supraorbital	2.0	20	1	4 weeks	No added effect of tDCS vs. sham
Choi et al., 2014 [29]	MPS	21	DLPFC/Contralateral	2.0	20	5	Post 5 sessions	Greater pain reduction in DLPFC group
Belley et al., 2018 [28]	Rotator Cuff Tendinopathy	40	M1/Contralateral	2.0	20	10	12 weeks	No additional benefits of tDCS
Sakrajai et al., 2014 [30]	MPS	31	M1/Contralateral	1.5	20	5	1 and 4 weeks	Improved pain and ROM in tDCS group

**Table 3:** Summary of tDCS effects on pain and function by cortical target and clinical condition.

Clinical Condition	Cortical Target	Pain Effect	Function Effect
Myofascial Pain Syndrome (MPS)	DLPFC	✓	✓
Myofascial Pain Syndrome (MPS)	M1	✓	✓
Subacromial Pain Syndrome (SAPS)	M1	✗	✗
Rotator Cuff Tendinopathy	M1	✗	✗

✓ = significant improvement; ✗ = no significant improvement.

**Table 4:** Baseline and post-treatment pain levels (VAS) in included studies.

Study	Diagnosis	Group	Baseline VAS (Mean ± SD)	Post-Treatment VAS (Mean ± SD)	Pain Reduction (Mean)
Choi et al., 2014 [29]	Myofascial Pain Syndrome	Active tDCS	4.22 ± 0.52	2.56 ± 0.73	↓ 1.66
		Sham	4.37 ± 0.48	3.50 ± 0.63	↓ 0.87
Sakrajai et al., 2014 [30]	Myofascial Pain Syndrome	Active tDCS	6.5 ± 1.2	3.2 ± 1.0	↓ 3.3
		Sham	6.4 ± 1.3	5.1 ± 1.1	↓ 1.3
Larrivée et al., 2021 [27]	Subacromial Pain Syndrome	Active tDCS	6.8 ± 1.0	3.3 ± 0.9	↓ 3.5
		Sham	6.7 ± 1.1	4.5 ± 1.0	↓ 2.2
Belley et al., 2018 [28]	Rotator Cuff Tendinopathy	Active tDCS	~5.5 ± 1.0	~3.5 ± 1.2	↓ ~2.0
		Sham	~5.5 ± 1.0	~3.5 ± 1.2	↓ ~2.0

## Supplementary Materials

The following supporting information can be downloaded at https://www.mdpi.com/article/10.3390/brainsci15060584/s1: File S1: supplementary.
