# Supplementary material for: The Role of Transcranial Direct Current Stimulation in Chronic Shoulder Pain: A Scoping Review
Source: Brain Sci. 2025 May 28;15(6):584. doi: 10.3390/brainsci15060584 (PMC12191168; doi:10.3390/brainsci15060584)
Supplement: Supplementary file 1 [file brainsci-15-00584-s001.zip › brainsci-3649021-supplementary.pdf]

MEDLINE (via PubMed)

(("transcranial direct current stimulation"[MeSH Terms] OR "transcranial direct current stimulation"[tiab] OR tDCS[tiab]) AND ("shoulder pain"[MeSH Terms] OR "shoulder pain"[tiab] OR "rotator cuff injuries"[MeSH Terms] OR "rotator cuff"[tiab] OR "rotator cuff tendinopathy"[tiab] OR "subacromial pain syndrome"[tiab] OR "subacromial impingement"[tiab] OR "myofascial pain syndrome"[MeSH Terms] OR "myofascial pain"[tiab] OR MPS[tiab]) AND ("pain management"[MeSH Terms] OR "rehabilitation"[MeSH Terms] OR "physical therapy modalities"[MeSH Terms] OR "functional recovery"[tiab] OR "disability"[tiab] OR "shoulder function"[tiab]))

Cochrane CENTRAL

("transcranial direct current stimulation" OR tDCS) AND ("shoulder pain" OR "rotator cuff disorder" OR "rotator cuff injuries" OR "rotator cuff tendinopathy" OR "subacromial pain syndrome" OR "subacromial impingement" OR "myofascial pain syndrome" OR MPS) AND ("pain reduction" OR "functional recovery" OR "rehabilitation" OR "shoulder disability")

Scopus

TITLE-ABS-KEY("transcranial direct current stimulation" OR tDCS) AND TITLE-ABS-KEY("shoulder pain" OR "rotator cuff" OR "rotator cuff tendinopathy" OR "subacromial pain syndrome" OR "subacromial impingement" OR "myofascial pain syndrome" OR MPS) AND TITLE-ABS-KEY("pain management" OR "pain reduction" OR "rehabilitation" OR "functional recovery" OR "physical therapy" OR "shoulder disability")

PEDro (Physiotherapy Evidence Database)

Title/Abstract: "transcranial direct current stimulation" OR tDCS AND

Problem: shoulder pain OR rotator cuff OR subacromial pain OR myofascial pain

Web of Science (WoS)

TS=("transcranial direct current stimulation" OR tDCS) AND TS=("shoulder pain" OR "rotator cuff" OR "rotator cuff tendinopathy" OR "subacromial pain syndrome" OR "subacromial impingement" OR "myofascial pain syndrome" OR MPS) AND TS=("pain management" OR "functional recovery" OR rehabilitation OR "physical therapy" OR "shoulder disability")
